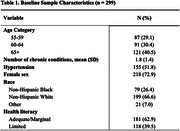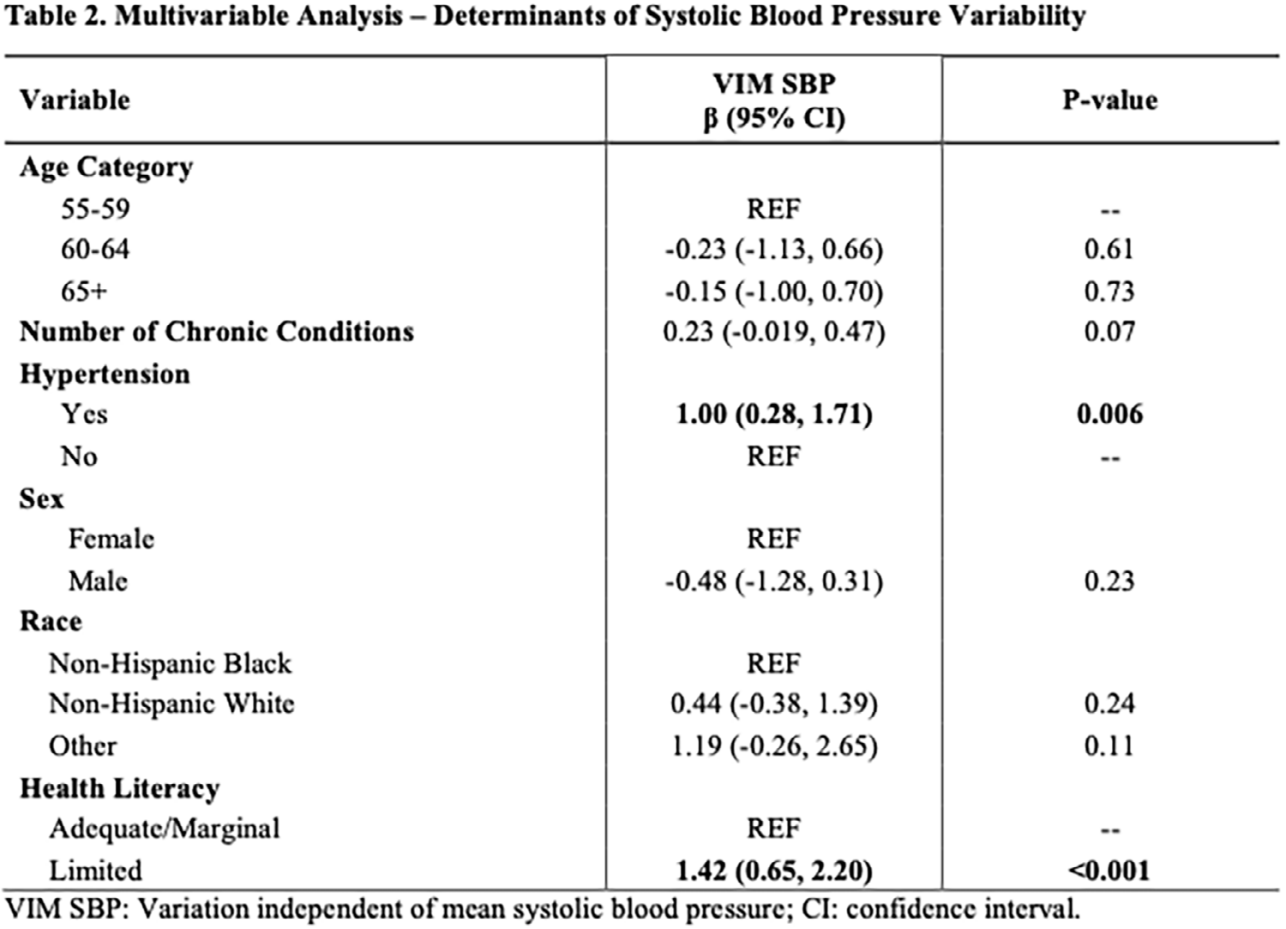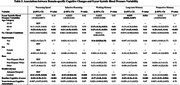# Health Literacy, Blood Pressure Variability, and Domain‐specific Cognitive Changes

**DOI:** 10.1002/alz.088947

**Published:** 2025-01-09

**Authors:** Keiko Ihara, Lauren Opsasnick, Morgan Bonham, Stephanie Batio, Julia Yoshino‐Benavente, Laura M Curtis, Stacy C Bailey, Michael S Wolf, Minjee Kim

**Affiliations:** ^1^ Japanese Red Cross Ashikaga Hospital, Ashikaga, Tochigi Japan; ^2^ Keio University School of Medicine, Shinjuku, Tokyo Japan; ^3^ Northwestern University Feinberg School of Medicine, Chicago, IL USA

## Abstract

**Background:**

Both limited health literacy (HL) and elevated blood pressure variability (BPV) in later life have been associated with the risk of dementia and cognitive impairment. However, little is known about the relationship between HL, BPV, and domain‐specific cognitive decline. We aimed to examine this relationship among primary care older adults.

**Method:**

English‐speaking adults aged 55‐74 were recruited from an academic general internal medicine practice and federally qualified health centers in Chicago between 8/2008 and 6/2010. HL was measured by the Newest Vital Sign. BPV was assessed by variation independent of mean systolic blood pressure calculated from all blood pressure measures obtained during routine ambulatory visits that occurred in calendar years 2008 – 2012. Five cognitive domains (processing speed, inductive reasoning, and working, long‐term, and prospective memories) were assessed through 15 tests at baseline, which were repeated three additional times at a 2.5‐year interval. Changes in Z‐scores were calculated from baseline (T1) to the last available assessment (T4 or T3) for each of the five cognitive domains. We examined associations between BPV and domain‐specific cognitive changes with univariate and multivariable regression, adjusting for a priori covariates including baseline age, sex, race, hypertension, number of chronic conditions excluding hypertension, cognitive function, HL, and interval between cognitive assessments.

**Result:**

A total of 299 participants (age 63.3 ± 5.3; 73% female; 26% non‐Hispanic Black, 67% non‐Hispanic White; 1.8 ± 1.3 chronic conditions; 51.8% with hypertension at baseline) were included in analyses (Table 1). Median interval between cognitive assessments was 8.4 years (IQR 8.2‐8.7). Baseline hypertension (β, 1.00; 95% confidence interval (CI), 0.28‐1.71; p = 0.006) and limited HL (β, 1.42; CI, 0.65‐2.20; p<0.001) were significant predictors of 5‐year BPV (Table 2). Greater 5‐year BPV was significantly associated with decline in long‐term memory (β, ‐0.035; CI, (‐0.064)‐(‐0.007); p = 0.016), but not with changes in other cognitive domains, after adjusting for a priori covariates (Table 3).

**Conclusion:**

Limited health literacy in later life is associated with greater variability of systolic blood pressure over five years, which was, in turn, associated with greater decline in long‐term memory over a median follow‐up of eight years.